# A road surface reconstruction dataset for autonomous driving

**DOI:** 10.1038/s41597-024-03261-9

**Published:** 2024-05-06

**Authors:** Tong Zhao, Yichen Xie, Mingyu Ding, Lei Yang, Masayoshi Tomizuka, Yintao Wei

**Affiliations:** 1 Tsinghua University, School of Vehicle and Mobility, Beijing, 100084 China; 2 University of California Berkeley, Department of Mechanical Engineering, Berkeley, CA 94709 USA

**Keywords:** Mechanical engineering, Computer science

## Abstract

Recent developments in intelligent robot systems, especially autonomous vehicles, put forward higher requirements for safety and comfort. Road conditions are crucial factors affecting the comprehensive performance of ground vehicles. Nonetheless, existing environment perception datasets for autonomous driving lack attention to road surface areas. In this paper, we introduce the road surface reconstruction dataset, providing multi-modal, high-resolution, and high-precision data collected by real-vehicle platform in diverse driving conditions. It covers common road types containing approximately 16,000 pairs of stereo images, point clouds, and ground-truth depth/disparity maps, with accurate data processing pipelines to ensure its quality. Preliminary evaluations reveal the effectiveness of our dataset and the challenge of the task, underscoring substantial opportunities of it as a valuable resource for advancing computer vision techniques. The reconstructed road structure and texture contribute to the analysis and prediction of vehicle responses for motion planning and control systems.

## Background & Summary

Environment perception lays the foundation for motion planning and control systems of unmanned robots and ground vehicles^1,2^. The progress of autonomous vehicle (AV) perception is always promoted by the emergence of large-scale datasets. Diverse multi-modal datasets have been published in the past decade, such as KITTI^3^, Argoverse^4^, and nuScenes^5^. The 3D surroundings and semantic information can be recovered by advanced deep learning models based on multi-modal data^6,7^.

Despite the remarkable strides on both datasets and algorithms^8–10^, they typically focus on above-road traffic perception by segmentation, object detection, and tracking. The road surface conditions, particularly road friction and unevenness parameters, are frequently overlooked or simplistically treated as constant constraints. According to the U.S. Federal Highway Administration (FHWA), in 2020 there are 32.11% unpaved roads in urban and rural areas^11^, which account for up to 20% of fatalities in some states^12^. About 15% of all road crashes are caused by low road friction such as wet pavement^13^. The road surface, being the sole interface with which vehicles establish physical contact, essentially determines the safety and comfort boundaries of vehicle dynamics^14–16^. The precision and performance of control systems are inherently limited without the knowledge of road surface. Therefore, besides traffic environment understanding, road surface perception remains a critical bottleneck in ensuring overall AVs performance.

Road reconstruction, aiming at recovering fine-grained road profile and texture with camera or LiDAR sensors, is an emerging topic in the technical stacks of AVs^17,18^. It significantly benefits predicting vehicle response in advance thus enabling proactive decisions to avoid potential safety risks^19–21^. Although many reconstruction research with available datasets have been reported^22,23^, the accuracy and fineness are insufficient for real-vehicle applications as the datasets provide sparse information for road surfaces. First, images in these datasets hold small areas for road surface, leaving low definition especially at far distances due to the perspective effect^24^. Then, neither accuracy nor density of LiDAR labels is adequate. Unlike traffic objects such as pedestrians and vehicles with large scale, road unevenness like rocks and cracks generally have small amplitudes^25^. Most datasets utilize LiDAR sensors with accuracy of ±3 cm, which is incapable of capturing accurate road profile variations. Furthermore, existing datasets are generally captured in cities with structured roads, whose scenario coverage is insufficient. Recovering detailed road profiles from these datasets is not promising.

Above all, there are hardly unified and comprehensive datasets to develop and evaluate road reconstruction applications. To solve the problems and fill this gap, we transfer the perception perspective from traffic scenarios to roads. This work presents a road surface reconstruction dataset named RSRD^26^, which to the best of our knowledge, is the first large-scale and real-world dataset special for road surface reconstruction. Note that 63 point cloud frames in this dataset are utilized in our previous work^27^, which focus on point cloud segmentation. None of the rest data in this dataset has been publicly reported.

**Fig. 1:**
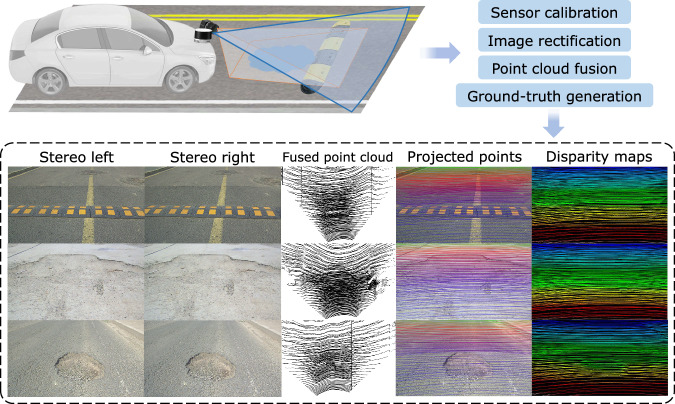
Schematic overview of the study.

Figure 1 shows the schematic overview and data samples of this study. For real-vehicle data acquisition, we build a hardware platform containing stereo camera, LiDAR, IMU, and RTK sensors. Both the camera and LiDAR concentrate on forward road surface rather than the whole traffic surrounding. Fine road textures and dense road point clouds are retained. Experiments are conducted in urban and rural areas covering diverse road surface conditions. Raw data undergoes calibration, rectification, motion compensation, and fusion procedures to create the RSRD. It outperforms the other datasets by providing about 16,000 pairs of high-resolution and high-accuracy road stereo images, point clouds, ground-truth depth/disparity maps, and vehicle motion information. Our RSRD can serve as an effective benchmark for extensive tasks encompassing vision or LiDAR-based reconstruction, localization, and mapping.

Our dataset represents a pioneering contribution toward promoting autonomous driving by road surface reconstruction. It may contribute to both research and applications in terms of (**i**) developing universal 3D vision methods like monocular depth estimation, stereo matching, and multi-view stereo; (**ii**) exploring point cloud processing and motion estimation algorithms for robots and vehicles; (**iii**) estimating road unevenness and friction from reconstructed road profile and texture thus benefiting vehicle safety and comfort control systems; (**iv**) road crack monitoring for pavement maintenance.

## Methods

In this section, we comprehensively describe the methodology utilized to build this dataset, including data acquisition platform, experiment design, data pre-processing and post-processing pipelines for multi-modal data.

### Hardware platform

**Fig. 2:**
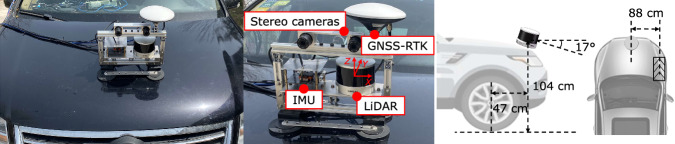
An illustration of the hardware platform.

**Fig. 3:**
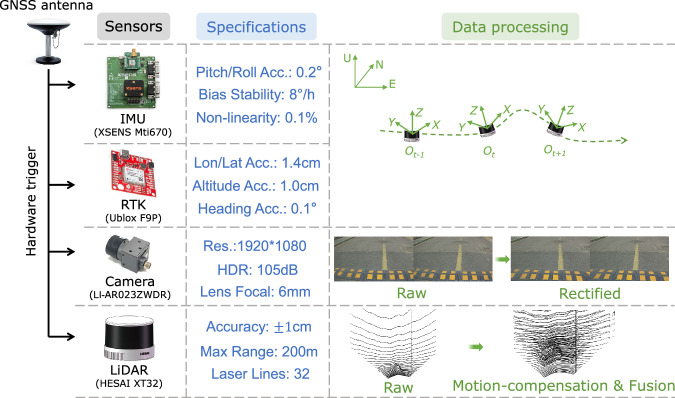
Sensor specifications and data processing.

Figure 2 shows the developed hardware platform, while Fig. 3 shows the sensor specifications and the corresponding data processing methods. Unlike the common sensor installation, the suit is mounted on the bonnet with a 17° pitch angle for prototype purposes. The perspectives of camera and LiDAR sensors focus more on the road area rather than the whole surrounding. The suit consists of a 32-line LiDAR, two cameras, a IMU, and a RTK system. The typical accuracy and precision of the LiDAR are ± 1 cm and 0.5 cm, respectively, which are higher than most of these adopted in existing datasets. It can capture mild road undulations and damages, ensuring high-precision road perception. Since we consider only the road surface area, the horizontal viewing angle of the mechanical rotating LiDAR is set to 100°.

The cameras generate clear and sharp images with dynamic range up to 105 dB, guaranteeing imaging quality in severe brightness changes. It has inside algorithms that prevent ghost blur in multi-exposure HDR imaging. The two cameras are fixed by a designed rigid holder with 12 cm baseline. The road preview distances of the cameras are about 14 m. The IMU and the RTK antenna are placed near the LiDAR to measure its orientation and position. We established a temporary fixed basement to achieve more stable and reliable localization results. The position and pose measurements are utilized in the following multi-frame point cloud fusion. The cameras and LiDAR run at 5 Hz, so the LiDAR can acquire more points in one frame. The IMU collects orientation data at 400 Hz, while 10 Hz as for the RTK. All the sensors are hardware-synchronized by the Pulse Per Second (PPS) from GNSS. The cameras start exposure when the LiDAR exactly rotates to the forward position. All the data samples have timestamps in UTC format. The sensors are integrated with the aluminum profile framework and tightly fixed to ensure a rigid connection.

The sensors are calibrated separately to ensure comprehensive accuracy. The stereo camera and the camera-LiDAR extrinsic parameters are calibrated with high-precision checkerboards. Specifically, the two cameras are first calibrated using a checkerboard with 12*9 square grids each of 2 cm size. Camera intrinsic parameter, lens distortion coefficients, rotation and translation matrices between the two cameras are derived. We utilize the Stereo Camera Calibrator in Matlab (https://www.mathworks.com/help/vision/camera-calibration.html) to achieve this, which implements the calibration method in^28^. The extrinsic parameter between left camera and LiDAR is calibrated statically with another checkerboard with 6*7 square grids each of 8 cm. We adopt the Lidar Camera Calibrator (https://www.mathworks.com/help/lidar/lidarcameracalibration.html) to calculate the calibration parameter. The overall re-projection error is smaller than 1 pixel.

### Experiment and data collection

Experiments are conducted from March to April, 2023 in Beijing and Qingdao, China. Driving on uneven roads results in severe vibration of the vehicle body. Therefore, the vehicle velocity is limited to under 40 km/h to prevent image motion blur and achieve denser road scan. Raw data is collected on concrete and asphalt roads in urban and rural areas with various uneven conditions, covering about 30 km of roads. We specially pick road segments with representative characteristics like bumps, potholes, continuously uneven surfaces, and texture-less areas. The acquired data covers common conditions for passenger vehicles, providing a valuable benchmark to dive into practical road image patterns.

The sensors are connected to a IPC running Python environment. Sensor working conditions and data flow are managed and collected by a script, where each sensor corresponds to an independent process. The two cameras, simultaneously triggered by the 5 Hz PPS, transmit YUV image data by USB protocol. The left and right images are compressed and saved in *.jpg* format with saving quality of 100. The LiDAR data is delivered by Ethernet protocol, which are then decoded to point cloud with the provided software kit. Frames including *xyz* coordinate values are saved in .*pcd* format. Each point has its timestamp at micro-second precision, which can be synchronized with other measured data. The IMU measures the roll and pitch angles of ego motion, which are sent to the host machine by CAN bus. The RTK module outputs longitude, latitude, altitude (LLA), heading (i.e., yaw) and velocity information. All the raw orientation, location, and velocity signals along with timestamps are saved in.*txt* files.

### Motion information processing

Motion information is essential for sequence-based applications including the point cloud fusion below. Positions originally measured by RTK module include LLA in WGS84 coordinate. The longitude and latitude are presented in *ddd.ddddddd*° format. The altitude is the height over mean sea level in mm unit. The LLA can be converted into relative translation in the local East-North-Up (ENU) frame considering the earth geometry model. The raw pose signals are transformed to the LiDAR coordinate, i.e., describing LiDAR’s rotation w.r.t. the local ENU coordinate. The definition of heading angle in our settings is: 0° when the vehicle faces south, while increase to 360° when rotating counterclockwise from bird’s eye view. The pitch and roll are rotation angles w.r.t the *X* and *Y* axes respectively, obeying the right-hand rule. The rotation sequence is yaw-pitch-roll in intrinsic rotations (rotated axis). The corresponding pose of the cameras can also be derived with the LiDRA-camera extrinsic. The horizontal velocity values are also provided in the local East-North coordinate.

### Image rectification and point cloud fusion

For eliminating the image distortion caused by imperfect installation and lens, the stereo images are rectified using the OpenCV functions (https://docs.opencv.org/4.2.0/d9/d0c/group__calib3d.html). After rectification, the corresponding point in one image can be found on the same row of another image. The column difference of corresponding pixels is defined as disparity, which is the target of stereo matching.

The single-frame LiDAR point cloud is still sparse, making fine-grained reconstruction challenging. Multi-frame fusion is required to accumulate nearby points^29^. We give a general description here as the detailed theoretical deduction is introduced in our previous work^27^. First, the points in nearby 46 frames are aligned to the same origin with the motion information, which is actually the motion compensation. Specifically, the translation and pose variation in the local ENU coordinate relative to the origin are interpolated for all points to be fused, after which the points are compensated and transformed into the original LiDAR coordinate. Then, the Iterative Closest Point (ICP) registration algorithm and its improved forms^30^ are utilized to further refine the fusion. The earlier and later frames are registered to the origin frame. To avoid extra noise and guarantee the dataset quality, we manually fine-tune the ICP hyper-parameters by grid-search for every sample and pick the one with the highest alignment accuracy. Note that the position accuracy of RTK may decrease due to the multi-path effect. Therefore, the point cloud fusion above is implemented only to frame segments with 1.4 cm localization precision.

**Fig. 4:**
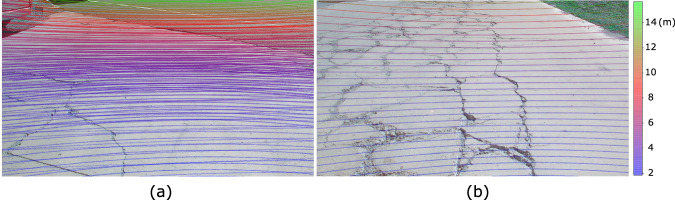
Comparison of projected point cloud between the (**a**) multi-frame fused and (**b**) single-frame. The color bar indicates depth.

Figure 4 shows the fused and single-frame point clouds projected onto images. The point cloud density is significantly promoted after fusion, making it superior for supervised learning requiring ground-truth labels. The average alignment errors in the road surface’s horizontal and vertical directions are bounded by ±1.2 cm. This error level guarantees the preservation of detailed road surface unevenness such as slight cracks and rocks.

### Ground-truth labels

Supervised learning requires massive data with ground-truth labels to fit models. Since this dataset emphasizes the reconstruction of road surface, we provide road profile geometry labels converted from point clouds. We do not offer semantic annotations like segmentation masks and detection bounding boxes, as they are inappropriate for this task. Practical road conditions are variable and therefore, in most cases, it is hard to clearly distinguish the foreground and background. Road unevenness like cracks and continuously uneven surfaces have no regular patterns or shapes, while segmentation or detection are insufficient to describe the complicated road profiles.

To generate the ground-truth depth maps, the road surface point clouds are first projected onto the rectified left image plane by using the LiDAR-camera extrinsic, while only the points within camera’s perspective are preserved. The *z* coordinate values are the depth of corresponding pixels. Depth maps with the same resolution (i.e., 1920*1080) are obtained, which serve as labels for methods like monocular depth estimation, structure from motion, and multi-view stereo. For stereo matching algorithms, ground-truth disparity *d* is derived according to the relationship *d* = *f*·*b*/*z*, where *z* is the depth value, *f* and *b* are the camera focal length in pixel unit and stereo baseline, respectively. The preview distance of the cameras is about 14 m, resulting in disparity values between 20–140 for the full-resolution stereo images.

### Methods for technical validation

For technical validation of this RSRD, we perform two typical computer vision tasks to reconstruct road surface: monocular depth estimation and stereo matching. For prototype purpose, we adopt the full-resolution (1920*1080) images and dense label maps to test the usability and reliability of the dataset.

#### Monocular depth estimation

We adopt seven depth estimation algorithms that ever achieved the state-of-the-art (SOTA) performance and re-implement their provided codes on our RSRD. The full-resolution images of the left camera are taken as inputs, while the depth maps are utilized as supervision to fit models. The maximum depth is set as 14 meters. The models are trained for ten epochs for fair comparison. The batch size is set to fully utilize the memory of a RTX 3090 GPU. All the other parameters adopt default configurations in codes. We select the following commonly utilized metrics in depth estimation to evaluate the models: *Abs. Rel*. (the absolute relative error between actual and predicted depth values), *RMSE* (the root mean square error), *RMSE log* (the log of RMSE), and *Sq. Rel*. (the squared relative error).

#### Stereo matching

We select five stereo matching methods to fit the dataset. The stereo pairs are center-cropped to 1400*700 since stereo matching for 2 M resolution images burdens memory and computation in our test environment. The maximum disparity value is set as 128 for the cropped images. The five models are trained for five epochs. We evaluate the model performance with the following metrics: end point error (EPE) calculated as the average absolute disparity error, *n*-pixel percentage defined as the ratio of pixels with disparity errors bigger than *n*.

## Data Records

The dataset is available in both the data repositories^31–33^ and dataset webpage https://thu-rsxd.com/rsrd. For the convenience of organization and download, the dense and sparse subsets are stored in different repositories. In this section, we describe the detailed contents and file directory of RSRD.

### Dataset organization

Multi-frame point cloud fusion requires much human effort as the optimal registration parameters involve human selection. We finally build 2,793 pairs of samples with fused dense point cloud labels. Furthermore, to enlarge the dataset scale and scenario diversity, we provide another independent sparse subset containing about 13,000 data pairs with motion-compensated single-frame point cloud labels, as illustrated in Fig. 4b. Models trained on the dense subset will be more accurate and reliable for road reconstruction. Nonetheless, the two subsets are equivalent for applications that do not utilize depth or disparity supervision such as structure from motion. The sparse subset can also be used to pre-train deep learning models since its scale and pattern coverage are larger.

Among the two subsets, we extract some time-continuous sequences for motion-related applications. The time duration is 8 seconds for each sequence, indicating 40 samples as the data acquisition frequency is 5 Hz. The aforementioned motion information is attached for every sample in the sequences. There are 15 and 176 sequences in the dense and sparse subsets, respectively.

Moreover, despite image resolution at 1920*1080 preserves fine road surface texture, it requires much memory and computation thus posing challenges for developing deep learning models. Therefore, we provide the down-sampled images and label maps with half resolution 960*540 for both the two subsets. The original and down-sampled sets share the same point clouds and motion information since they are independent from image resolution. Researchers can determine which resolution to utilize according to their preferences.

### Dataset directory

**Fig. 5:**
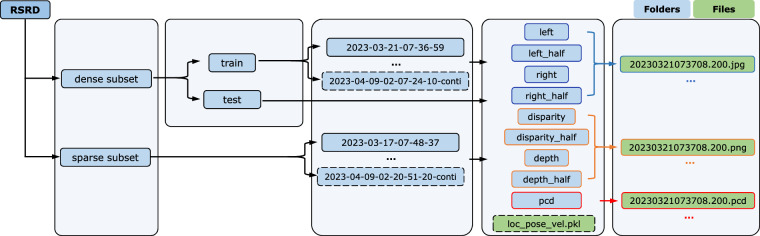
Folder directory of the dataset.

The dataset is compressed into one.*zip* file, whose folder directory is shown in Fig. 5. All the multi-modal data samples are normatively formatted for convenient usage. All files are named by the corresponding timestamp of 5 Hz trigger in *YYYYMMDDHHmmSS.sss* format, e.g., *20230408042202.800.jpg, 20230317074852.200.pcd*. Data in one day shares the same calibration file, which can be indexed by the date in their timestamps. The calibration files, including camera intrinsic, stereo baseline, and left camera-LiADR extrinsic parameters, are provided in the development kit described in the *Code Availability* section.

For the convenience of model development and fair model performance comparison, we further split the dense subset into train set with 2,493 pairs, and test set with 300 pairs. Data samples in the train set are placed into folders named in format *YYYY-MM-DD-HH-mm-SS*, e.g., *2023-03-21-07-36-59*. There are no content difference of the folders, but indicating that data in these folders are acquired near the time declared by the folder names. Table 1 enumerates the number of data pairs in the folders of train set. Note that the 15 continuous sequences are all in the train set and settled in 15 separate folders with ‘*conti*’ in folder name. Motion states at every moment of these sequences are saved in binary files named *loc_pose_vel.pkl*, which are directly placed in the corresponding root folders. The sequences are not listed in Table 1 as their number of data pairs are always 40.

**Table 1:** Folder-wise counts of sample pairs in train split of the dense subset.

Folder name	Number of data pairs
2023-03-17-07-48-37	133
2023-03-21-07-36-59	67
2023-04-06-01-38-49	166
2023-04-08-02-33-11	96
2023-04-08-03-04-21	134
2023-04-08-03-15-19	167
2023-04-08-03-26-11	223
2023-04-08-04-21-42	100
2023-04-08-04-46-16	62
2023-04-09-01-57-56	88
2023-04-09-02-07-24	517
2023-04-09-02-20-51	140

Multi-modal data are placed in sub-folders named by the corresponding data type. The *left* and *right* folders store stereo left and right images, respectively. The *depth* and *disparity* indicate the ground-truth depth and disparity maps w.r.t. the left camera, respectively. Point clouds are saved in *pcd* folders. Folders with *half* in name store the down-sampled images and label maps with half resolution, i.e., 960*540. File names in the nine folders are the same excluding file extensions. Therefore, the target data at the same moment can be indexed by the file names.

The sparse subset has the same directory structure as the dense subset without being split into train or test sets. Table 2 also shows the number of data pairs in the folders of sparse subset.

**Table 2:** Folder-wise counts of sample pairs in train split of the sparse subset.

Folder name	Number of data pairs	Folder name	Number of data pairs
2023-03-17-07-48-37	399	2023-04-08-04-21-42	221
2023-03-21-07-36-59	184	2023-04-08-04-38-49	186
2023-04-06-01-38-49	218	2023-04-08-04-46-16	184
2023-04-06-01-42-50	468	2023-04-08-04-47-43	142
2023-04-08-02-33-11	638	2023-04-08-04-49-08	384
2023-04-08-03-15-19	225	2023-04-09-01-57-56	202
2023-04-08-03-18-09	427	2023-04-09-02-00-22	903
2023-04-08-03-26-11	142	2023-04-09-02-07-24	1138
2023-04-08-04-03-42	149	2023-04-09-02-20-51	422

### Interpretations about data format

The stereo images are saved in .*jpg* format with saving quality of 100. The depth and disparity maps are saved in 16-bit.*png* format. Values in maps without ground-truth label are set as 0. The actual depth or disparity values can be obtained by dividing 256. The .*pcd* files in the final dataset contains only *xyz* fields of points. The.*pkl* files storing motion information are generated by the *pickle* lib in Python, which can be parsed with the function in our development kit.

## Technical Validation

In this section, we first perform thorough statistic analysis on our RSRD from many aspects. Results prove that our dataset outperforms the others in terms of road surface reconstruction applications. Results of the technical validation algorithms in *Methods* section are presented and explained.

### Comparison with existing datasets

To demonstrate the superiority of our RSRD, we comprehensively compare the existing vision datasets with stereo images for AVs perception as shown in Table 3. The widely used KITTI dataset^3,34^ contains few samples in the stereo subset, based on which the performance of deep learning models to be developed cannot be ensured. The DrivingStereo^35^ has much more samples by collecting data in similar scenarios and road sections. The *road ratio* indicates the ratio of road area to the whole image. The existing datasets have low road ratios since they care the complete traffic environment. The *GT ratio* is the percentage of pixels with ground-truth LiDAR points. Nevertheless, this metric is not directly comparable since it can be improved by reducing the image resolution. Our RSRD still reaches 4.12% even at 1920*1080 resolution, while 17.08% for 960*540 resolution. The ApolloScape^36^ achieves extremely dense labels by fitting CAD models to cars and roads. Recovering the actual road profiles is almost impossible since the road surfaces are regarded as planes.

**Table 3:** Comparison of the existing datasets with stereo images for AVs perception.

	# samples	Resolution	B (cm)	F (px)	LiDAR acc. (cm)	Road ratio (%)	GT ratio (%)	Disp. acc. (px)
KITTI’12^3^	389	1242 × 375	54	719	± 2	18.3	28.04	0.5
KITTI’15^34^	400	1242 × 375	54	719	± 2	20.6	19.72	0.6
Argoverse^4^	6624	2464 × 2056	29.7	3757	± 3	31.6	0.78	0.7
ApolloScape^36^	5165	3130 × 960	—	—	± 0.5	30.1	78.24	8.2
DrivingStereo^35^	182188	881 × 400	54	2061	± 2	37.7	21.18	1.0
KAIST Urban^39^	—	1280 × 560	47.5	775	± 3	32.2	—	—
FordAV^40^	—	1656 × 860	52.9	945	± 2	16.0	—	—
Oxford Robot^41^	—	1280 × 960	24	983	± 3	29.3	—	—
RSRD(Ours)	2793 + 13672	1920 × 1080	12	2022	± 1	89.1	4.12	0.6

The *B* indicates stereo baseline, and *F* is the camera focal length. We randomly extract 100 samples from every dataset, and evaluate the *Road ratio*, *GT ratio*, and *Disparity accuracy* metrics on them. The *KAIST Urban*, *FordAV*, and *Oxford Robot* datasets do not directly provide the rectified stereo images.

We also assessed the average *disparity accuracy*, which is a comprehensive evaluation involving all the errors in sensor acquisition, fusion, and calibration processes. We pick corresponding pixels at different positions of the stereo images and calculate their errors between the LiDAR-measured disparity values. Our RSRD achieves an error of 0.6, which is generally equivalent to the KITTI. It outperforms all the other datasets as for the corresponding depth error because our cameras have a smaller value of focal multiplying baseline. The human-designed model fitting causes significant disparity errors in the ApolloScape. Also, the errors are inconsistent among samples, possibly because of the temporary loss of RTK. The Argoverse-stereo has higher errors at object boundaries, possibly because of poor motion compensation or joint calibration.

The road condition diversity of the compared datasets is relatively poor as they focus on the whole traffic condition. The accuracy and label density do not satisfy the requirements of precise and dense road surface perception applications. By comprehensive comparison, our RSRD has superiority among all metrics and is a better alternative for road surface perception.

### Analysis of point cloud label density

**Fig. 6:**
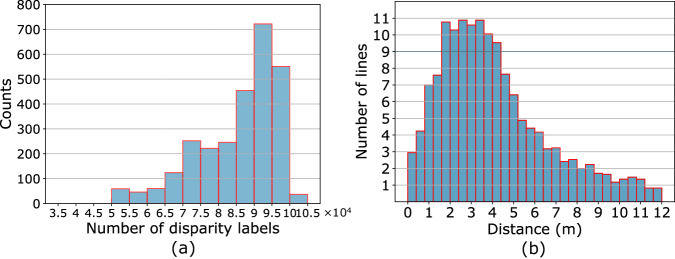
Density of point cloud labels. (**a**) histogram of the number of disparity labels. (**b**) number of LiDAR scanlines in 40 cm intervals along the longitudinal direction of road surface.

We count the number of label points of left images in the dense subset, and the histogram is shown in the left sub-figure of Fig. 6. Most image samples have 70K–100K pixels with ground-truth depth and disparity values, corresponding to the ratio between 3.4%–4.8% for full-resolution images while 13.6%–19.2% for half-resolution images. Also, we evaluate the point density along the longitudinal direction of road surface. The number of LiDAR scanlines in intervals of 40 cm is counted, as shown in the right sub-figure of Fig. 6. Within preview distance of 6 meters, averagely at least one scanline per 10 cm can be ensured. The reconstruction performance is expected to decrease from 7 meters away since both the ground-truth density and road surface definition are low.

### Validation results of monocular depth estimation

**Fig. 7:**
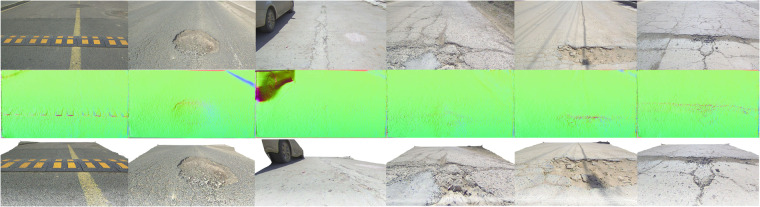
Inference results by monocular depth estimation methods. From up to down: input RGB images, surface normal maps, and colored point clouds. For better visualization, we show the surface normal maps calculated from the depth maps.

**Fig. 8:**
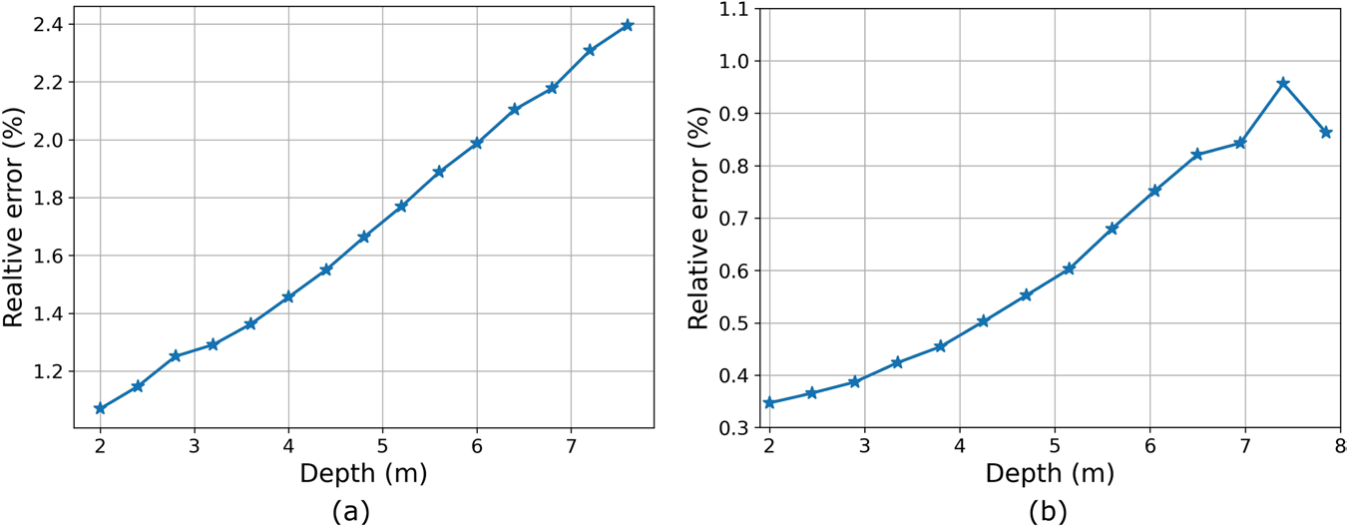
Visualization of depth-wise relative error. The depth interval is set as 40 cm. (**a**) results from AdaBins. (**b**) results from ACVNet.

Figure 7 visualizes monocular depth estimation results derived by the AdaBins model^37^. For better visualization, we convert depth maps into normal maps since depth can not obviously present the slight road unevenness. The speed bump, potholes and cracks are precisely recovered, verifying the effectiveness of our RSRD in capturing road surface structures. Benefiting from the high accuracy and dense point cloud labels, all the models achieve distinguished values on the metrics in Table 4. However, the average relative depth error around 2% indicates an absolute error of 10 cm at 5 m depth. The accuracy is inadequate for practical applications since road surface vibrations are generally smaller than this level. Further, as shown in Fig. 8, we visualize the depth-wise relative error in the range of 2~8 m with interval of 40 cm. The relative error increases with depth, indicating higher accuracy at near distance. This phenomenon is consistent with the data pattern as texture details are retained at small depth while lost at large depth because of the perspective effect. The dataset is quite challenging and therefore, leaves much space for researchers in developing advanced models to achieve more accurate estimation.

**Table 4:** Evaluation results with monocular depth estimation methods.

Method	Abs Rel ↓	RMSE ↓	RMSE log ↓	Sq Rel ↓
AdaBins^37^	0.016	0.150	0.023	0.005
NeWCRFs^42^	0.033	0.294	0.044	0.017
BTS^43^	0.019	0.172	0.026	0.006
SAN^44^	0.029	0.219	0.036	0.009
iDisc^45^	0.019	0.174	0.026	0.006
PixelFormer^46^	0.019	0.176	0.026	0.006
LapDepth^47^	0.023	0.217	0.032	0.009

### Validation results of stereo matching

The test metrics including EPE, 1-pixel, and 3-pixel ratios are listed in Table 5. The disparity errors of all models are around 0.4 pixels, which is at the sub-pixel level. More than 95% of pixels have the estimation error less than 1 pixel. Considering the camera intrinsic and extrinsic parameters, the average disparity error corresponds to a depth error of 4 cm at 5 m depth. Also, we convert disparity into depth and visualize the depth-wise relative error as shown in Fig. 8. Stereo matching also presents the increasing trend of error with respect to depth. However, the magnitude is smaller than that of monocular depth estimation. For instance, the relative error at 2.5 m depth is 0.37%, translating to an absolute error of 0.9 cm. Recovering road profiles by stereo cameras is expected to be more promising than the monocular.

**Table 5:** Evaluation results with stereo matching methods.

Method	EPE (px)	>1 px (%)	>3 px (%)
RAFT-Stereo^48^	0.450	8.139	1.157
ACVNet^49^	0.354	4.885	0.100
IGEV-Stereo^50^	0.369	4.896	0.151
CFNet^51^	0.333	3.276	0.063
GwcNet^52^	0.412	5.890	0.255

We preliminarily validate the dataset with existing algorithms by adopting the full-resolution images. For faster training and model development, researchers can utilize the half-resolution images.

## Usage Notes

The motion information of sequences is provided in primary form, i.e., Euler angles and LLA. We provide function that converts the LLA to relative translation. Researchers can also convert them into required formats such as extrinsic parameters of adjacent frames or these relative to the first frame. If required, the depth maps of right images can also be generated as point clouds and calibration parameters are all provided.

This dataset is mainly for road reconstruction purpose based on vision or point cloud. We do not provide semantic-related labels such as segmentation and detection. Researchers can make corresponding annotations on images or points for supervised learning.

The dataset, released with license CC BY 4.0, is open to download.

## Data Availability

We provide a development kit programmed with Python language for this dataset, which contains scripts for visualizing and parsing the dataset. The toolkit is available at the code repository^38^ (https://github.com/ztsrxh/RSRD_dev_toolkit). The *projection.py* provides functions for reading calibration parameters, reading disparity and depth maps, projecting points onto images and pixels onto points, as well as their visualization. The *read_imu_rtk.py* shows an example that parses the motion information and convert them into relative location under ENU coordinate. The *data_reader.py* implements the *Dataloader* in PyTorch that provides training samples. The *cam_extrinsic.py* implements the calculation of camera extrinsic parameter between two time clocks. The extrinsic is presented as the translation and rotation matrices from the current time to the origin. The code has MIT license for unrestricted usage.
